# High‐Throughput Metal 3D Printing Pen Enabled by a Continuous Molten Droplet Transfer

**DOI:** 10.1002/advs.202205085

**Published:** 2022-12-16

**Authors:** Chan Kyu Kim, Dae‐Won Cho, Seok Kim, Sang Woo Song, Kang Myung Seo, Young Tae Cho

**Affiliations:** ^1^ Department of Mechanical Engineering Changwon National University 20, Changwondaehak‐ro, Uichang‐gu Changwon‐si Gyeongsangnam‐do 51140 Republic of Korea; ^2^ Department of Joining Technology Materials Testing & Reliability Division Korea Institute of Materials Science 797, Changwon‐daero, Seongsan‐gu Changwon‐si Gyeongsangnam‐do 51508 Republic of Korea; ^3^ Busan Machinery Research Center Korea Institute of Machinery and Materials 48, Mieumsandan 5–40, 41beon‐gil, Gangseo‐gu Busan 46744 Republic of Korea

**Keywords:** 3D printing, arc plasma, continuous freeway additive manufacturing, high throughput, metal 3D pen

## Abstract

In metal additive manufacturing (AM), arc plasma is attracting attention as an alternative heat source to expensive lasers to enable the use of various metal wire materials with a high deposition efficiency. However, the stepwise material deposition and resulting limited number of degrees of freedom limit their potential for high‐throughput and large‐scale production for industrial applications. Herein, a high‐throughput metal 3D printing pen (M3DPen) strategy is proposed based on an arc plasma heat source by harnessing the surface tension of the molten metal for enabling continuous material deposition without a downward flow by gravity. The proposed approach differs from conventional arc‐based metal AM in that it controls the solidification and cooling time between interlayers of a point‐by‐point deposition path, thereby allowing for continuous metal 3D printing of freestanding and overhanging structures at once. The resulting mechanical properties and unique microstructures by continuous metal deposition that occur due to the difference in the thermal conditions of the molten metal under cooling are also investigated. This technology can be applied to a wide range of alloy systems and industrial manufacturing, thereby providing new possibilities for metal 3D printing.

## Graphical abstract

The arc plasma‐based M3DPen is capable of additively manufacturing line‐shaped metal structures by controlling the volume of molten droplets. Controlling the droplet volume and the solidification volume of the molten metal allows the surface tension to prevent the molten metal from flowing down due to gravity. Continuous and free metal AM is possible without requiring support and long cooling time.

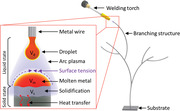

## 1 Introduction

Metal additive manufacturing (AM) is rapidly emerging as a new manufacturing process that allows for the fabrication of complex 3D objects of metal or alloys such as impellers,^[^ ^1^, ^2^ ^]^ turbine blades,^[^ ^3^, ^4^ ^]^ propellers,^[^ ^5^, ^6^, ^7^ ^]^ and lattice structures.^[^ ^8^, ^9^, ^10^ ^]^ The commonly used metal AM technique is based on arc plasma heat sources such as gas metal arc welding(GMAW) or gas tungsten arc welding(GTAW) and exhibits various advantages, including low manufacturing costs, high productivity,^[^ ^11^, ^12^ ^]^ and dissimilar deposition.^[^ ^13^, ^14^, ^15^ ^]^ Moreover, recent advances have substantially supported the use of various wire materials such as aluminum,^[^ ^16^, ^17^, ^18^ ^]^ titanium,^[^ ^19^, ^20^, ^21^ ^]^ and nickel alloys.^[^ ^22^, ^23^, ^24^, ^25^ ^]^ However, the path generation in the arc‐based metal AM is still based on a point‐by‐point or layer‐by‐layer manufacturing approach.^[^ ^26^, ^27^, ^28^ ^]^ Thus, their print speed is limited by stepwise material deposition due to the time of solidification and cooling of molten metals during the printing process.^[^ ^29^, ^30^, ^31^, ^32^ ^]^ This is because conventional arc‐based metal 3D printing processes typically require solidified beads as supporting material to attach the molten metal droplet and to form a stable structure. In addition, an increasing heat input for a high deposition rate excessively increases the volume of molten metal during the printing process and causes it to flow downward owing to gravity (Figures S1–S3, Supporting Information).^[^ ^33^ ^]^ Consequently, the cooling time in between the stepwise material deposition is a critical parameter for control of the printing throughput correlated with the solidification of the molten that affects the continuity of the deposition process and the overall productivity.

In this work, we introduced a continuous metal 3D printing pen (M3DPen) technology that can resolve this throughput issue in arc‐based metal AM by balancing the surface tension and solidification of the molten metal beads. This proposed technology is a nonstop process fundamentally different from traditional arc‐based metal AM techniques in which heat input, molten bead formation, and solidification/cooling of molten metal need to be conducted separately stepwise. We demonstrated that a continuous bead formation along with the deposition path during the metal solidification can be implemented by harnessing the surface tension of molten metal drops, thereby forming a stable 3D structure in a similar way to a 3D printing pen of polymer materials.^[^ ^34^, ^35^ ^]^ In addition, we investigated the resulting mechanical properties and unique microstructures of the continuous 3D printed metal structures with different thermal conditions of the molten metal compared with that of the conventional arc‐based metal AM process. Furthermore, although the conventional metal printing process based on powder bed fusion or direct energy deposition typically requires a support structure or material to form a stable structure, the proposed approach can manufacture freestanding or overhanging structures without these supports at once. In particular, the supports require extensive energy and time for removing materials or structures using various physical methods;^[^ ^36^ ^]^ thus, our method that involves the continuous deposition of metal could provide considerable advantages in the metal AM field. The M3DPen process presented herein is expected to promote the throughput of large‐scale metal 3D products adequate for the manufacturing industry.

## 2 Results and Discussion

### 2.1 Mechanism of M3DPen

**Figure 1:**
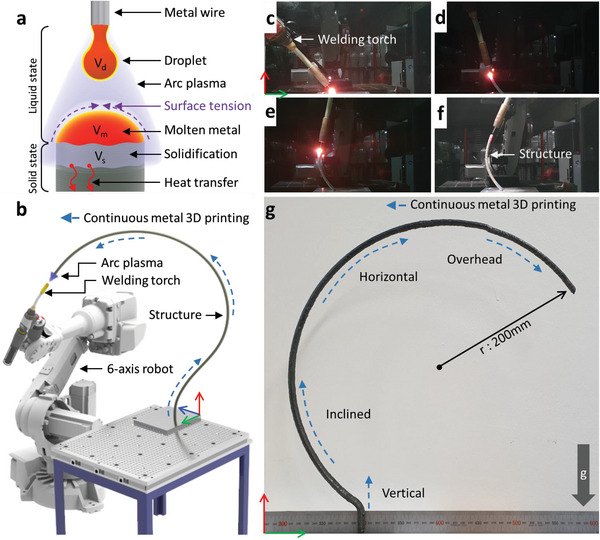
Schematic and optical image of the M3DPen process. a) Scheme of the metal additive manufacturing system for M3DPen. b–e) Sequence of the M3DPen process for a curved structure without support (Movie S1, Supporting Information). f) Metal structure in the form of circles. g) Continuous metal addition is possible in all directions, such as vertical, horizontal, and overhead, for which the welding torch moves in three dimensions.

**Figure 2:**
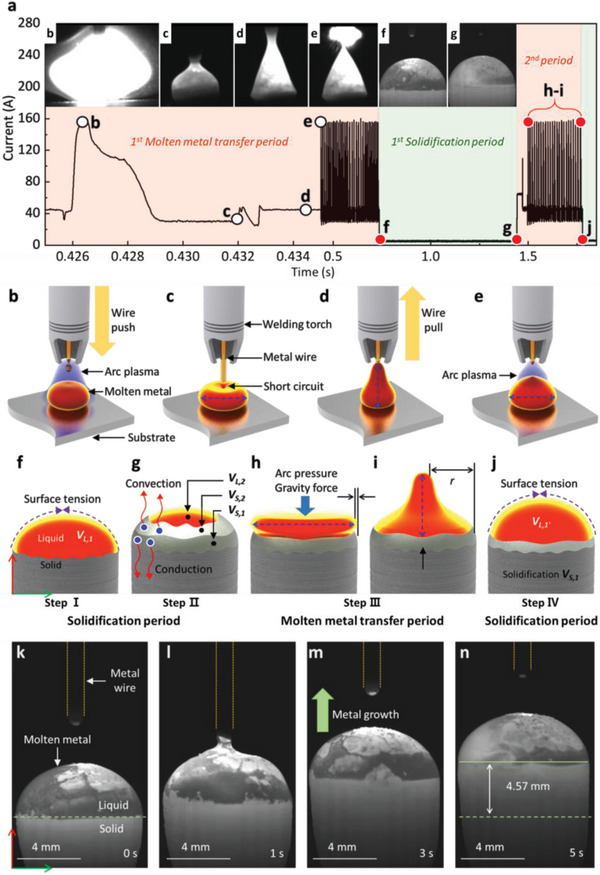
Typical schematic and optical image of the M3DPen process. a) Current waveform of the M3DPen process consisting of molten metal transfer period (red section) and solidification period (green section). b–e) Sequence of a single cycle of the molten metal transfer period. f–j) Sequence of the M3DPen process when molten metal transfer and solidification periods are repeated. VL,1 and VL,1’ refer to the volume of the molten metal immediately after the molten metal transfer is completed, and VL,2 is the reduced volume of the molten metal, which has decreased due to cooling from convection and conduction. VS,1 and VS,2 represent the solidified volume of the molten metal. k–n) High‐speed camera image of M3DPen process wherein molten metal transfer and solidification periods are repeated (Movie S2, Supporting Information).

**Figure** **1** shows a schematic of the proposed M3DPen process. Figure 1a shows the key strategy of M3DPen. The molten metal of volume *V*_m_ has its own surface tension, maintains a constant shape, and solidifies to a solid state over time. When the volume of the molten metal increases, its shape solidifies non‐uniformly due to gravity. Therefore, by adding droplet volume (*V*_d_) to solidify the molten metal (*V*_s_), constant *V*_m_ can be maintained during AM. As shown in Figure 1b, the proposed system for AM comprises a six‐axis robot and an arc plasma heat source. The desired structure can be fabricated by the M3DPen process with an Inconel 625 filler wire possessing high‐temperature corrosion resistance. Figure 1c–f displays the sequence images of the fabrication of a curved metal structure using M3DPen, which enables continuous metal deposition to obtain a 3D object with the desired shape without supports (Figure 1g; Movie S1, Supporting Information). **Figure** **2**a depicts the current waveform during the M3DPen process, and each period can be sectioned into molten‐metal transfer and solidification periods (Figure S4, Supporting Information). In the molten‐metal transfer period, as shown in Figure 2b–e, the current waveform and arc plasma on/off conditions change according to wire motion, such as pull and push motions (Figure S5, Supporting Information). As the feeding direction of the wire repeatedly varies between positive and negative, the molten metal fluctuates according to the capillary action of the wire, arc forces (arc pressure, electromagnetic force), and gravity. As this cycle repeats, the volume of the printed structure increases; however, solidification immediately occurs upon rapidly controlling the wire movement after the molten‐metal transfer period (Figure 2f–j;Figure S6 and S7, Supporting Information). Consequently, the volume of molten metal (*V*_L,1_) can be continuously and stably maintained by balancing its input and solidification, thereby enabling the formation of a stable liquid droplet owing to the surface tension. In detail, owing to the cooling caused by conduction and convection, solidification (*V*_S,1_ and *V*_S,2_) occurs from the bottom to the top of the molten metal, and the volume (*V*_L,1_ − *V*_L,2_) of the molten metal gradually decreases. Before the complete solidification of the molten metal, a short‐circuit metal transfer is performed to increase its volume and re‐melt its solidified volume (*V*_S,2_). Subsequently, wire motion, arc pressure, and gravity result in the fluctuating behavior of the molten metal. Finally, the volume (*V*_L,1’_) of the molten metal is maintained constant (*V*_L,1_ = *V*_L,1’_), and the solidified volume (*V*_S,2_) is deposited. As the molten‐metal transfer and solidification are repeated as shown in Figure 2f–j, the upper part of the fabricated structure is continuously formed owing to deposition in a liquid state with a constant volume, as shown in Figure 2k–n (Movie S2, Supporting Information).

### 2.2 Computational Analysis of M3Dpen

**Figure 3:**
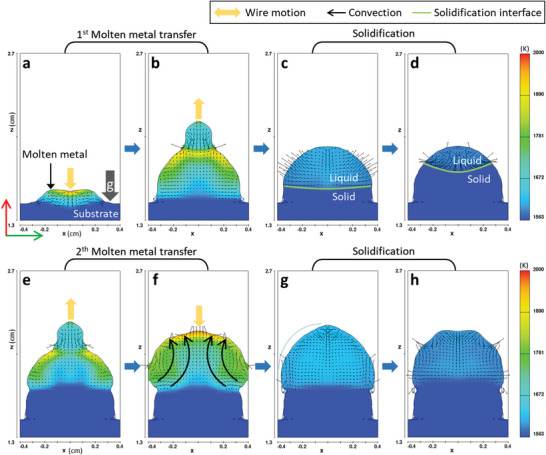
Temperature profiles and the velocity fields from transient CFD simulations for M3DPen process. a,b) Behavior of the molten metal during the first molten metal transfer. The volume of the molten metal increases, and the shape changes with the push and pull motions of the metal wire. c,d) Solidification of molten metal for heat transfer after the first molten metal transfer. Under surface tension, the molten metal solidifies as the solidification interface between liquid and solid increases while maintaining a semi‐elliptical shape. e,f) Behavior of the molten metal during the second molten metal transfer before complete solidification. The volume of the molten metal increases as the solidified volume is re‐melted due to molten metal transfer. g,h) Solidification of the molten metal for heat transfer after the second molten metal transfer.

To further understand the proposed M3DPen process, the solidification process of the molten metal formed during the M3DPen process was simulated through 3D transient computational fluid dynamics (CFD) using the volume‐of‐fluid technique, and the stable deposition of molten metal in the M3DPen process was proven. **Figure** **3** displays the 3D‐transient‐analysis results (Figure 2a: current waveform) for the deposition and solidification of molten metal in M3DPen. When the arc is on, the heat input from the arc heat source and molten‐metal transfer is applied to the simulation. The arc pressure, electromagnetic force, drag force, surface tension, gravitational force, and metal impingement that contribute to the momentum of the fluid were considered to simulate the molten‐metal behavior. However, because there is no heat input when the arc is off, the molten metal solidifies owing to heat conduction, whereas the surface tension and gravity only affect the fluid flow. Figure 3a,b depicts the behavior of the molten metal subsequent to the first metal transfer. The volume of the molten metal gradually increases as the shape of the molten metal changes horizontally and vertically owing to the motion of the wire. When the molten‐metal transfer is complete, the surface of the molten metal is solidified while maintaining a semi‐elliptical shape owing to the surface tension. As shown in Figure 3c,d, a phase change (liquid to solid) occurs from the bottom of the molten metal because of heat transfer owing to conduction and convection. Before the upper part of the molten metal is completely solidified, the second molten‐metal transfer starts, as shown in Figure 3e,f. The increased volume due to the molten‐metal transfer is the solidified volume due to heat transfer, as shown in Figure 3c,d. Following the second molten‐metal transfer, solidification occurs while maintaining a semi‐elliptical shape owing to the surface tension, as shown in Figure 3g,h. Therefore, if the above cycle is repeated, a stable M3DPen process can be achieved while maintaining a constant volume and shape of the molten metal.

### 2.3 Mechanical Properties and Microstructures of Continuous 3D Printed Metal Structures

**Figure 4:**
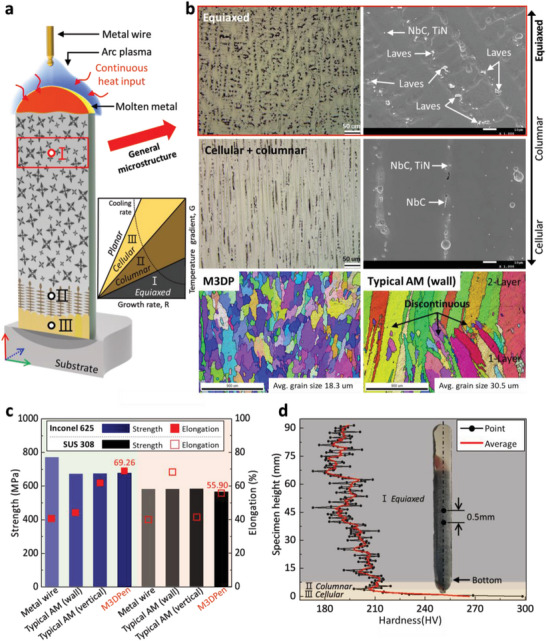
Thermal gradient and growth rate on the morphology of solidification microstructure and mechanical properties. a) Schematic of morphology solidification microstructure for thermal gradient and cooling rate. b) Optical, SEM, and EBSD images for microstructure and second phases. c) Comparison of tensile strength and elongation according to the deposition method. d Hardness graph measured at 0.5 mm intervals from bottom to top for a vertical specimen.

We investigated the morphology of the microstructure and the mechanical properties of metal structures manufactured using the M3DPen process (**Figure** **4**). A schematic of the temperature gradient, G, and growth rate, R, of the solidification microstructure for a vertical specimen is shown in Figure 4a. Evidently, cellular dendrites can be grown in the direction of the welding torch because the temperature gradient is high owing to the conduction through the substrate. However, when a continuous heat input is applied, the temperature gradient gradually decreases, and columnar grains grow; finally, equiaxed grains grow at the end of the AM process.^[^ ^37^ ^]^ The extent of cellular and equiaxed structure formation is constant, and as the length of the specimen increases, columnar grains grow in a single direction. Figure 4b presents the optical, scanning electron microscope (SEM), and electron backscatter diffraction (EBSD) images of the specimen cross‐section from the bottom to the top. The specimen microstructure consists of cellular, columnar, and equiaxed grains, and equiaxed grains form the general microstructure. The images show that the second phase is concentrated at the top of the specimen, where the metal addition process is finished. The second phase in the form of NbC and TiN, which can appear during the deposition of Inconel 625, occurred in the bottom and middle of the specimen, in addition to the Laves phase (Ni_2_Nb). The second phase ^[^ ^38^, ^39^ ^]^ that deteriorates toughness, fatigue strength, creep strength, and corrosion properties are concentrated at the top of the specimen because the process always tends to maintain the molten metal in a liquid state. Further, the components of the second phase, such as Ni and Nb with a low density, are suspended by the convection, surface tension, and buoyancy forces of the molten metal. A comparison between the EBSD images for M3DPen and typical AM (wall) reveals that M3DPen produces equiaxed grains and indistinguishable interlayers. By contrast, in the case of the typical AM (wall) process, the interlayers are clearly distinguishable, along with the microstructure in multi‐layer welding. The tensile strength and elongation comparison according to deposition method are shown in Figure 4c. The deposition specimens are metal wire, typical AM in wall form, typical AM in vertical form, and M3DPen. A typical AM in wall form is general wire arc additive manufacturing (WAAM) process, in which metal beads are additively manufactured in a layer‐by‐layer deposition method. A typical AM in vertical form is a voxel‐like, dot‐by‐dot deposition process, where the molten metal is fully solidified before the next layer is deposited (Figure S8, Supporting Information). The analysis of SUS308 metal wire was also compared using the same deposition method as aforementioned. In particular, the stress‐strain curve results of the specimens manufactured by the M3DPen are attached to Figure S9 (Supporting Information). Summarizing the results, the maximum yield strength was 343.91 MPa, and the ultimate strength was 677.01 MPa. Further, the elongation was 69.26%, and the reduction area was 87.75%. Figure 4d depicts the hardness‐test results for the vertical specimen. Regarding the measurement points, the hardness tests were performed on the center line of the specimen at 0.5 mm intervals. At the bottom of the specimen, a cellular grain, the highest hardness of 298.38 Hv, was observed, and, as the deposition height increased, the hardness decreased, and an average of 195.5 Hv was measured. At the initial molten‐metal transfer, the small volume of molten metal increases as it bounces up and down because of wire motion and arc pressure. Subsequently, when the volume of the molten metal becomes sufficiently large, it grows while maintaining a smooth surface with a constant diameter. The specimens fabricated by M3DPen are characterized by low hardness and high elongation, but the strength is similar to that of conventional WAAM.^[^ ^40^ ^]^ In M3DPen, the thermal gradient of the molten metal gradually decreases owing to the continuous heat input. Hence, although the same metal wire is used, it affects the sizes of the formed microstructures and grains, indicating a difference in mechanical properties.^[^ ^41^ ^]^

### 2.4 Continuous Manufacturing of Freestanding 3D Structures

**Figure 5:**
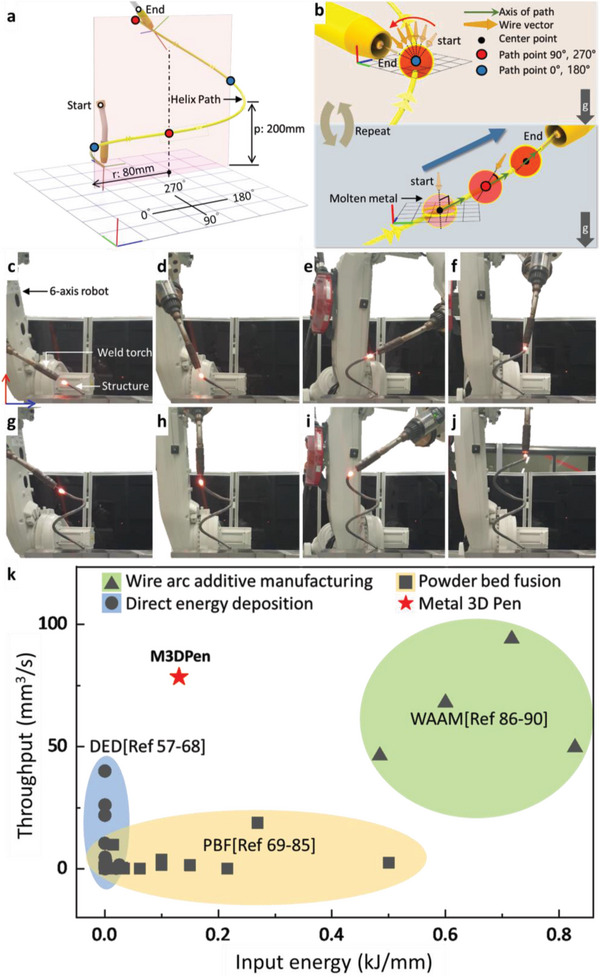
Helix structure fabricated by the M3DPen process without a positioner. a) Design of the deposition path for a helix structure. b) Mechanism of the deposition path and robot joint for the helix structure. c–j) Real‐time images of the additive manufacturing of a helix structure without a positioner (Movie S3, Supporting Information). k) Performance map of M3DPen process with single‐layer deposition (Table S1, Supporting Information).

Finally, the proposed M3DPen process allows for the continuous metal printing of freestanding 3D structures. **Figure** **5** shows a printed helix structure obtained by continuous metal deposition, which has a radius of 80 mm and a pitch of 200 mm. The mechanism of the path points from 0° to 270° for continuous manufacturing of the helix structure is shown in Figure 5b. At the path points at 90° and 270°, the welding torch is perpendicular to the reference plane and gradually becomes parallel to the axis of the deposition path. At the path points at 0° and 180°, the welding torch rotates 180° with respect to the axis of the deposition path. If the path from 0° to 270° is repeated, the helix structure can be continuously 3D‐printed without support and by only controlling the joint of the robot. Based on the mechanism shown in Figure 5b, the sequence of metal AM is shown in Figure 5c–j (Movie S3, Supporting Information). The welding torch has various vectors (not only parallel); however, it is possible to freely manufacture via deposition by controlling the solidification time according to the volume of the molten metal. In the case of simply using M3DPen, a lattice structure composed of a single wire or a spring‐like shape can be considered. However, if M3DPen is considered as an option in additive manufacturing, it becomes possible to design various deposition paths for structures. Figure S10 (Supporting Information) shows an image of a free‐form structure created by additive manufacturing using M3DPen and typical AM, in which a skeleton is first made with a single wire. Subsequently, a semicircle is deposited on the skeleton using a typical AM method. It is judged that additive manufacturing is possible after the deposition of a single wire and by proceeding with a new deposition. Further, a spline structure with a height of 600 mm and a step‐shaped structure are shown in Figure S11 (Supporting Information). Deposition can be performed as the larger the size of the robot used, the larger would be the size of the structure. After a single wire is formed, a new wire is successively deposited. Figure S12 (Supporting Information) shows the contouring method used instead of the existing layer‐by‐layer method when deposition is performed to create a propeller. The contouring method can improve the precision of the structure and serve as a support by making the outer line of the structure. The shape of the propeller is similar to the re‐entrant structure, and the width gradually increases as it goes up. Such a structure is difficult to create using additive manufacturing without support. A new metal deposition path using M3DPen is used when deposition is performed to create a propeller. First, the edges of the propeller are made using the contouring method (single wire). Contouring serves as a support while holding the basic frame of the shape. Thereafter, the interior is bulk filled using typical AM. Figure S12c (Supporting Information) shows an image of the actual propeller edge deposition using the contouring method. Although the curves of the propellers were flexibly deposited, additive manufacturing could not be carried out until the end owing to the mechanical limitations of the robot movement. However, limitation may be overcome by applying a positioner. Figure S13 (Supporting Information) shows an experiment in which the lower part of the propeller is deposited using M3DPen as a metal support. As aforementioned, the propeller has a shape that increases in width as it goes up. Creating precise shapes via the simple layer‐by‐layer method of WAAM is challenging. To overcome this problem, a new deposition path can be created by utilizing the single wire of M3DPen. Figure S13b (Supporting Information) shows an image of a single wire as a support. The previous additive manufacturing method using arc plasma entails deposition in a single pass by holding a welding torch vertically. However, it can serve as a support by making the outline of the shape as shown in the image. Thereafter, the interior can be bulk filled with the existing WAAM as shown in Figure S13c,d (Supporting Information). The series of experiments suggest the possibility to additively manufacture branching structures that cannot be realized with typical AM, for example, Figure S14 (Supporting Information). Figure 5k shows the performance chart for throughput‐input energy relationship in existing metal AM methods including DED (directed energy deposition), PBF (powder bed fusion), and WAAM. Here, the throughput and input energy are defined as the deposition volume per unit time for a single layer and heat being supplied to the metals to be melted respectively(Table S1, Supporting Information)^[^ ^42^, ^43^, ^44^, ^45^, ^46^, ^47^, ^48^, ^49^, ^50^, ^51^, ^52^, ^53^, ^54^, ^55^, ^56^, ^57^, ^58^, ^59^, ^60^, ^61^, ^62^, ^63^, ^64^, ^65^, ^66^, ^67^, ^68^, ^69^, ^70^, ^71^, ^72^, ^73^, ^74^, ^75^ ^]^. Thus, the upper left side in Figure 5k represents a more efficient method to enable high‐throughput printing at low input energy. In laser‐based metal AM technologies such as DED and PBF, the focused beam spot enables a high energy density at low heat input, thereby exhibiting clear advantages for a high cooling rate and precise material deposition process.^[^ ^41^ ^]^ However, the printing throughput is relatively low because the volume of material deposition is limited by the focused laser beam size. On the contrary, although the WAAM process enables a high deposition volume at high heat input due to the arc‐based metal AM method,^[^ ^76^ ^]^ the low cooling rate of molten metals and repetitive melting/solidification cycle impede the improvement of the overall printing efficiency. Our approach demonstrates the potential for overcoming the throughput‐input energy relationship compared with the conventional metal AM methods. The proposed M3DPen enables high‐throughput printing with low input energy because of the formation of a continuous liquid interface of molten metals and thereby prevents the use of excessive energy for the melting and repetitive long cooling time for the solidification.

## 3 Conclusion

Current efforts for the development of arc‐based metal AM technology are mainly focused on the manufacture of relatively simple 3D structures comprising a combination of straight lines, such as lattice structures,^[^ ^77^, ^78^, ^79^ ^]^ impellers,^[^ ^80^, ^81^, ^82^ ^]^ propellers,^[^ ^83^, ^84^, ^85^ ^]^ and turbine blades.^[^ ^86^, ^87^ ^]^ In addition, the issues of the deterioration of the shape precision and mechanical properties of prototypes under the cyclic melting and cooling of metal wires and the increased temperature of the structures due to the high heat input of the arc plasma remain unaddressed.

The M3DPen process developed in this study achieved continuous metal deposition while maintaining the upper portion of the molten metal in a liquid state without the discrete and repetitive melting and cooling of metal wires. Because of this continuous deposition of molten metal droplets, we also found that the metal structures manufactured by the M3DPen process exhibited a unique morphological characteristic compared with the discontinued layer‐by‐layer characteristic observed in conventional arc‐based metal AM, such as WAAM.^[^ ^88^ ^]^ We speculated that the gradual morphological change from cellular and columnar to equiaxed structures according to the location of the specimen may have originated from the internal temperature gradient in the continuously deposited molten metal. Nevertheless, further studies are required to analyze the effect of this temperature gradient on the microstructures or mechanical properties, considering that the molten metal remains in the liquid state owing to the continuous heat input. Moreover, to demonstrate the M3Dpen process in this study, only Inconel 625 metal wire with a diameter of 1.2 mm was used as a model material. We expect that this limitation will be addressed by performing further optimization using various metal wires, such as stainless steel or aluminum wires. In addition, the diameter of printed structure can be sufficiently controlled according to the arc plasma, size of the wire, and the metal wire feed rate shown in Figure S15 (Supporting Information).

To summarize, we believe that the M3DPen strategy presented herein would expand the potential and flexibility of metal 3D printing and increase the print throughput and degree of freedom beyond those of discrete pointwise printing in conventional arc‐based metal AM. For example, we have suggested one paradigm of increasing the degree of freedom in metal AM by rapidly manufacturing the outline of the structural skeleton of an overall 3D structure by the M3DPen process.^[^ ^89^, ^90^ ^]^ In this study, we demonstrated the continuous metal AM method by introducing the control of the arc‐off time and harnessing the surface tension of molten metal droplets so that the volume of the deposited molten metal can be regulated and maintained constant to form stable 3D structures. It is very difficult to use a six‐axis robot to create a deposition path due to restrictions such as the rotation radius and posture of the robot joints. For example, when performing additive manufacturing to create overhangs or re‐entrant structures, the robot will hit the ground. In addition, while manufacturing deposition structures such as knots and lattice structure, there is a risk that a torch or robot may collide with the structure. Although the physical mechanism for M3DPen was presented, there are limitations with regard to the design of its shape due to mechanical problems.

Further, even though the printing‐size range is limited in the current system, this metal printing strategy enables an innovative manufacturing approach for high‐throughput metal 3D printing.

## 4 Experimental Section

### System Preparation

The six‐axis robot (ABB IRB 6700, ABB, Switzerland) used had a payload of 200 kg and a maximum reach of 2600 mm. The arc plasma heat source (TPS 500i CMT, Fronius International, Austria) had a cold metal transfer (CMT) mode, and experimental conditions were as follows: current, 60 A; wire feed rate, 1.5 mpm; gas flow rate, 20 L min^−1^; arc length, 15 mm.

### Material Preparation

A Ø1.2 Inconel 625 metal wire (Kiswel, KW‐M625, Republic of Korea) and 99.95% argon shielding gas were used. The hot mounting resin (Polyfast, Struers, Denmark) was used for cross‐sectional observation of the specimen. The specimen was etched with methyl alcohol: nitric acid with a ratio of 17:3 (CH3OH:HNO3), and electrolytic etching (LectroPol‐5, Struers, Denmark) was performed for 75 s at 6 V.

### Mechanical Properties Analysis

Tensile test data (Hydraulic UTM, DAEKYUNG TECH, Republic of Korea) were obtained as a standard ASTM E8/E8M‐21. Hardness data were obtained using a micro‐indentation hardness tester with 200 gf HV, and a distance of 0.5 mm (LECO, LM‐248AT, USA).

### Characterization

The cross‐sectional images of the etched specimen were obtained from an optical microscope (Olympus, BX53M, Japan). SEM images were obtained using a field‐emission scanning electron microscope with an accelerating voltage of 10–20 kV (FE‐SEM/EDS, JSM‐7001F, JEOL, Japan). The behavior images of the molten metal were obtained using a high‐speed camera with 50–4000 fps (Photron, FASTCAM MINI AX100, Japan).

### CAD/CAM Preparation

The 3D CAD modeling for the generation of the deposition path was performed using design software (CATIA V5, DASSAULT system, France), and a path coordinate generation tool (ABB robot studio, ABB, Switzerland) was used.

### CFD Simulation

Molten metal analysis was conducted using commercial software (Flow‐3D version 9.3, Flow Science, USA) following the volume of fluid method. However, the authors developed the subroutines that affected the heat source and momentum for the M3DPen process.

## Conflict of Interest

The authors declare no conflict of interest.

## Author Contributions

C.K.Kim. and D.W.Cho. contributed equally to this work. C.K.Kim, S.Kim, and Y.T.Cho performed conceptualization. C.K.Kim, D.W.Cho, and Y.T.Cho performed methodology. C.K.Kim, S.W.Song, K.M.Seo, and S.Kim performed investigation. C.K.Kim, D.W.Cho, K.M.Seo, and S.Kim wrote the original draft. Y.T.Cho wrote the original draft and reviewed and edited the final manuscript. All authors discussed the results and commented on the paper.

## Supporting information

Supporting Information

Supplemental Movie 1

Supplemental Movie 2

Supplemental Movie 3

## Data Availability

Data sharing is not applicable to this article as no new data were created or analyzed in this study.
