# Supplementary material for: High‐Throughput Metal 3D Printing Pen Enabled by a Continuous Molten Droplet Transfer
Source: Adv Sci (Weinh). 2022 Dec 16;10(6):2205085. doi: 10.1002/advs.202205085 (PMC9951324; doi:10.1002/advs.202205085)
Supplement: Supplementary file 1 — Supporting Information [file ADVS-10-2205085-s001.pdf]

**Supporting Information**

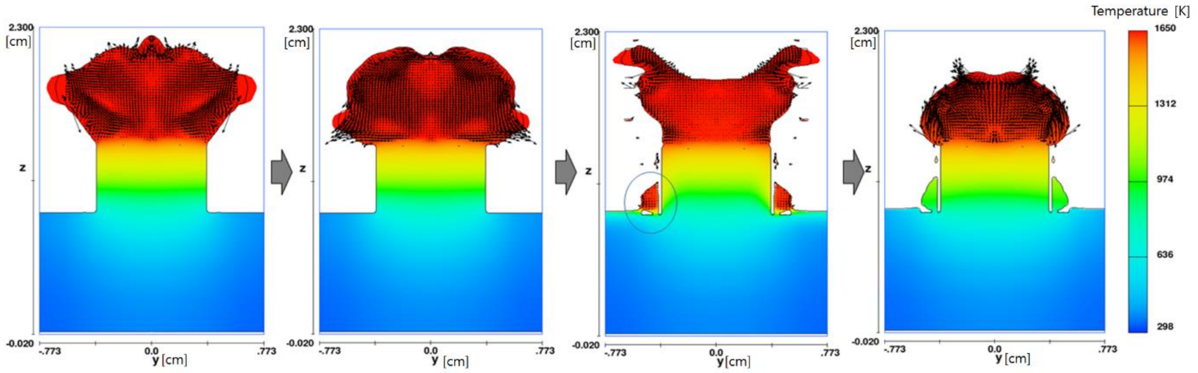


**Figure S1.** Molten metal formation process without arc – off time from the numerical simulation. If the arc-off time is not secured in the process, the continuous supplied molten metal makes the unstable formation and brings the molten metal overflows (dotted circle).

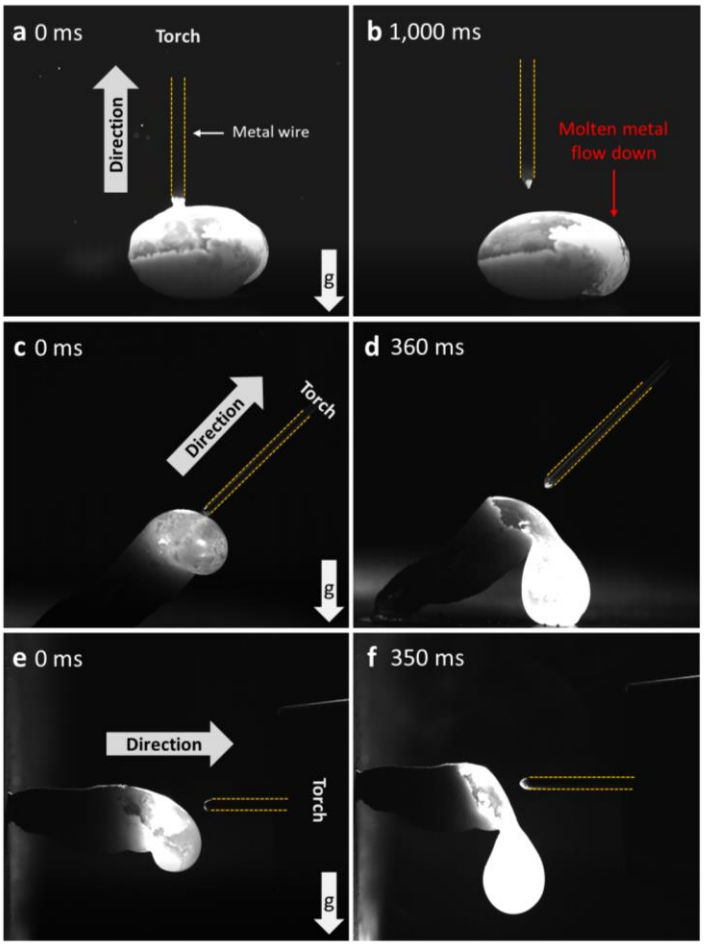


**Figure S2.** High-speed camera image of molten metal behavior according to deposition position. **a-b** Vertical position. **c-d** Inclined position. **e-f** Horizontal position. According to the printing posture, the flow time of the molten metal becomes faster.

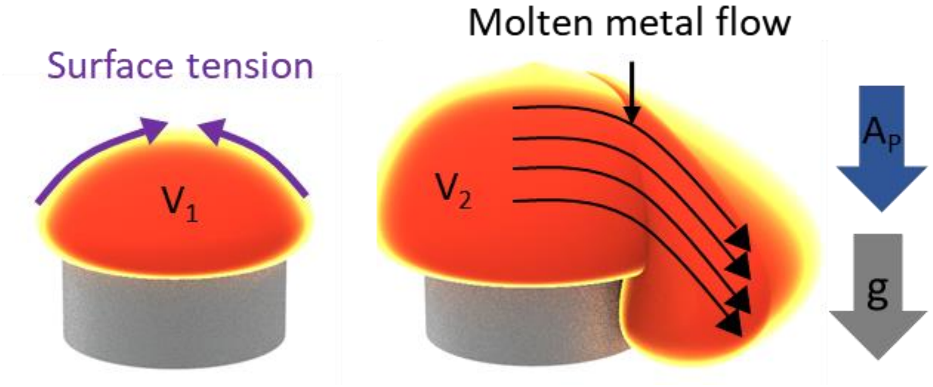


**Figure S3.** Mechanism of molten metal flow down. If the volume of molten metal in the liquid state is below the critical value, it solidifies while maintaining a constant semi-spherical shape due to surface tension. On the other hand, when the volume increases, the molten metal flows down in the direction of gravity due to an external force such as wire motion, arc pressure and gravity forces.

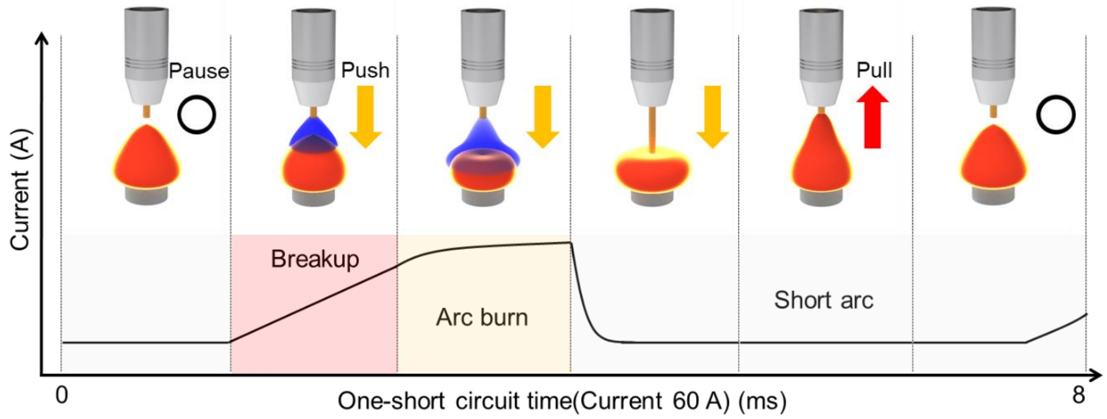


**Figure S4.** Current waveform according to one short circuit when current is 60 A.

Molten transfer proceeds with breakup, arc burn, and short arc and the sequence of processes takes approximately 8 ms.

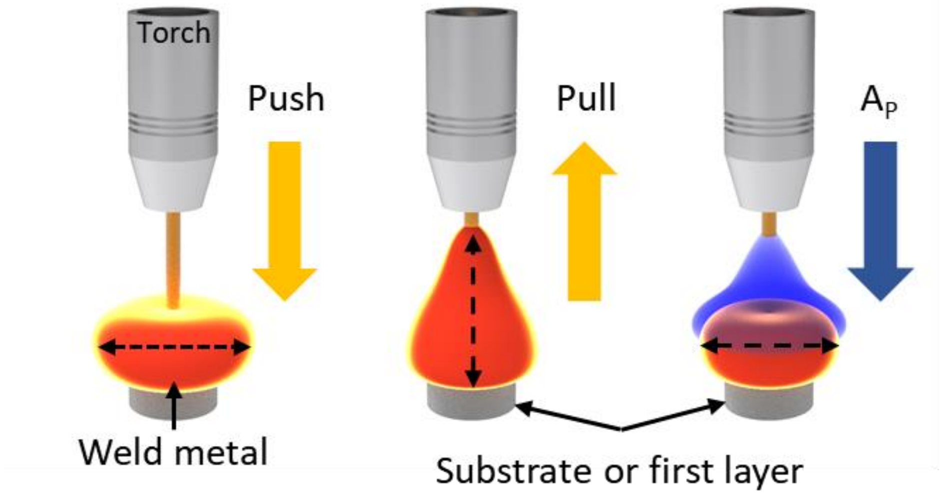


**Figure S5.** Behavior of molten metal according to wire motion. The CMT(cold metal transfer) mode, which is a short circuit transfer, mechanically controls the wire motion to proceed with the molten metal transfer. Molten metal is affected by wire motion, arc pressure and gravity forces.

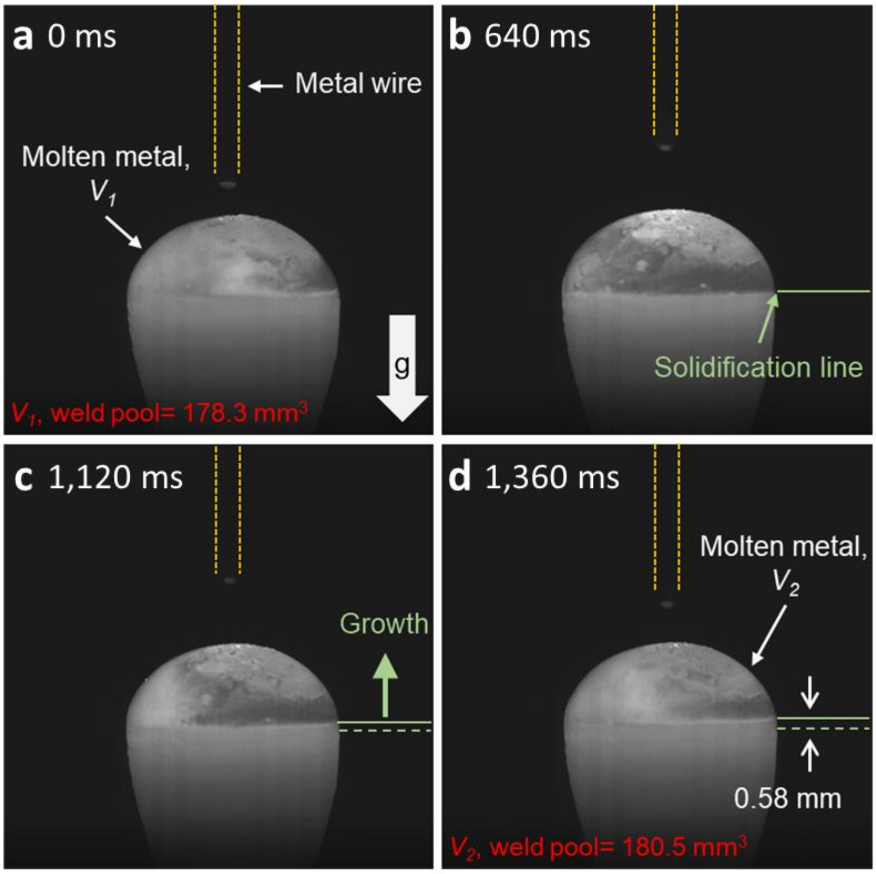


**Figure S6.** Additive manufacturing by controlling the volume of the molten metal **a** Before the first molten metal transfer at 0 ms. **b** After molten metal transfer for 640 ms. **c**

Solidification in progress for 1,120 ms and **d** Before the second molten metal transfer at 1,360 ms.

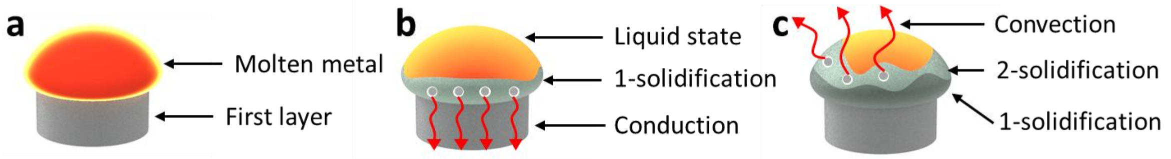


**Figure S7.** Schematic principle of molten metal solidification **a** Immediately after molten metal transfer cycle. **b** First solidification between bottom of the molten metal and the substrate due to conduction and **c** Second solidification of the surface of molten metal due to convection.

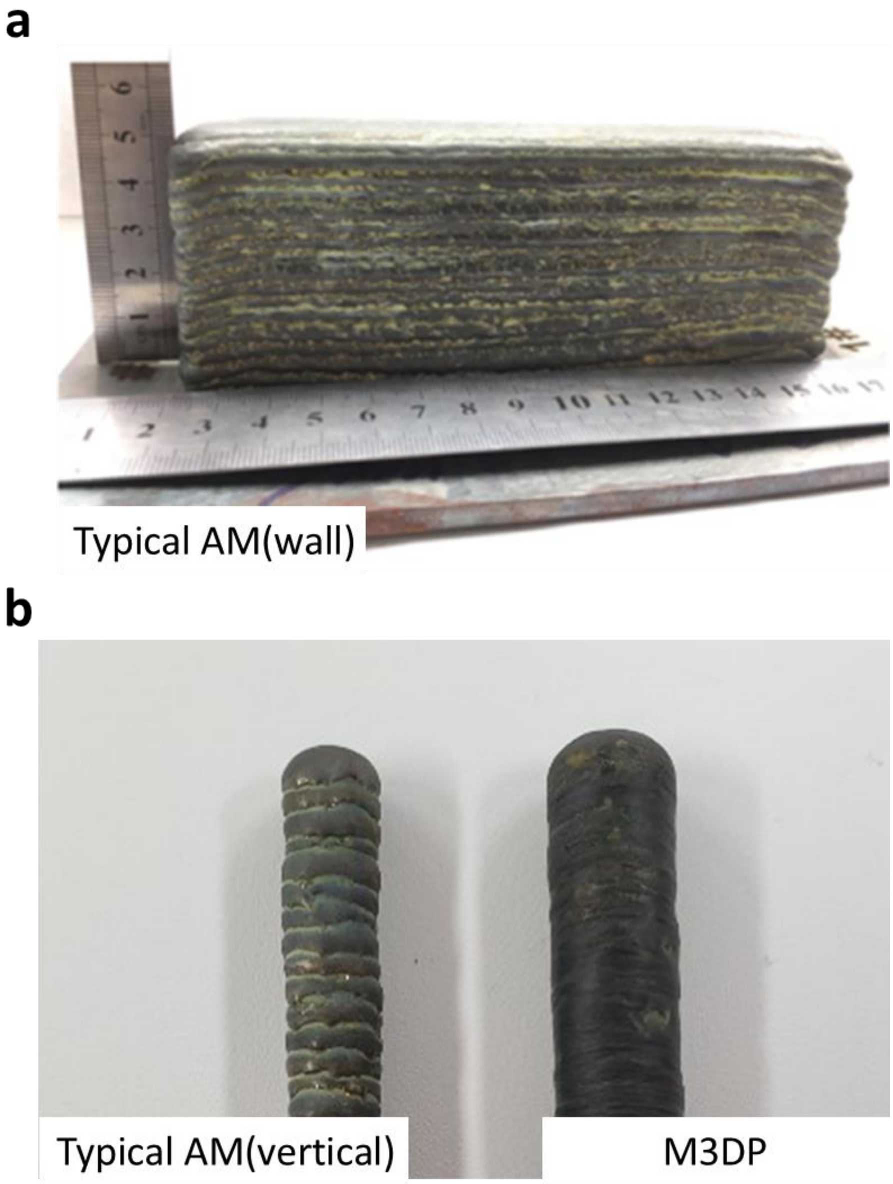

**Figure S8.** Example of additive manufacturing method for metal specimens. **a** Typical AM (wall) deposition method. A typical AM is a single bead deposition layer by layer by applying sufficient cooling time as in the existing WAAM. **b** L; Typical AM (vertical) deposition method that is fully solidified by dot-by-dot method, R; M3DPen deposition method in this study.

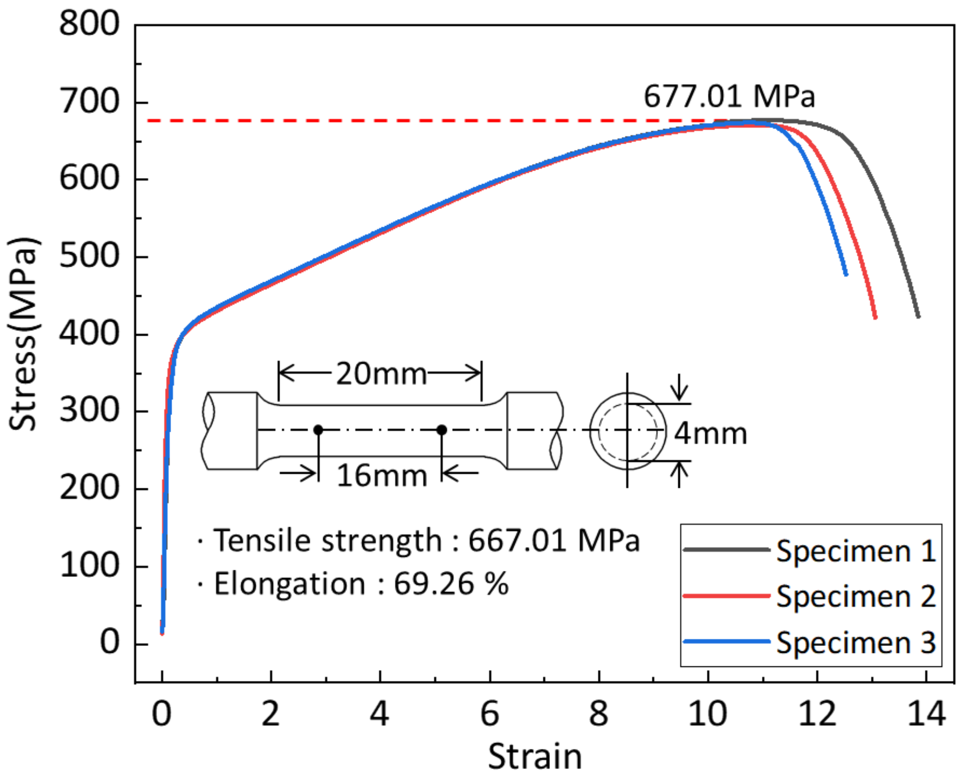


**Figure S9.** Stress-strain curve of the specimen fabricated by the metal 3D printing pen process. Tensile strength tests were performed on three specimens under the same deposition conditions using Inconel 625 metal wire.

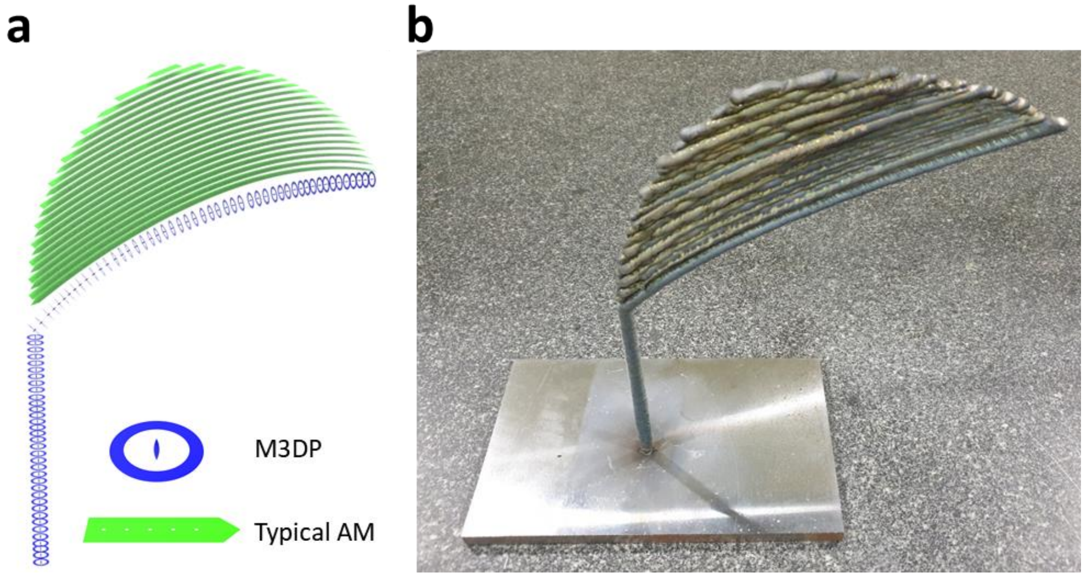

**Figure S10.** Metal free-form additive manufacturing using M3DPen and WAAM. **a** Modular path planning for free-form structure. **b** Metal additive manufacturing with modular path planning. A guide line was made with a single wire and the structure was made in a layer-by-layer deposition method. It is possible to create a deposition path differentiated from WAAM.

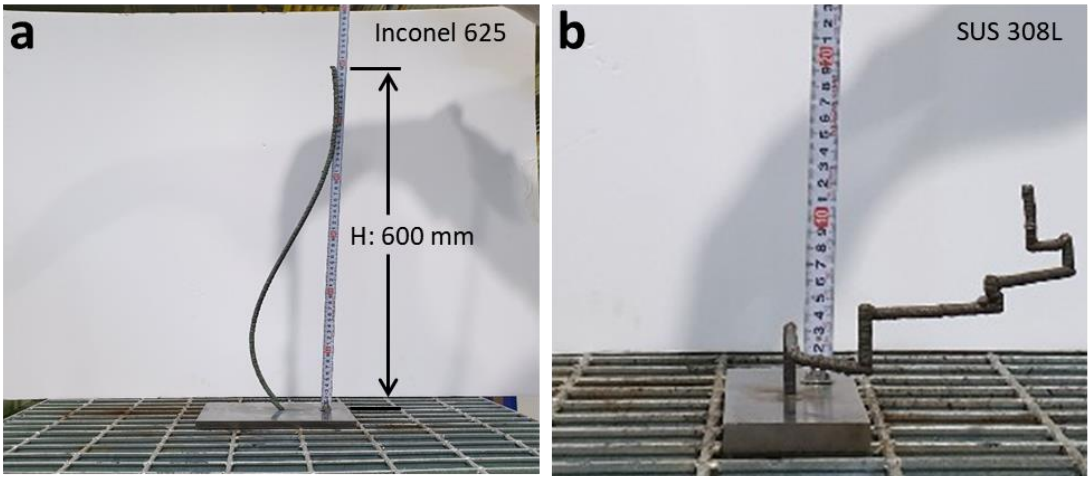


**Figure S11.** Additive manufacturing in the form of spline curves and steps. **a** Additive manufacturing of spline structures with a height of 600 mm. **b** Additive manufacturing of step-shaped structure.

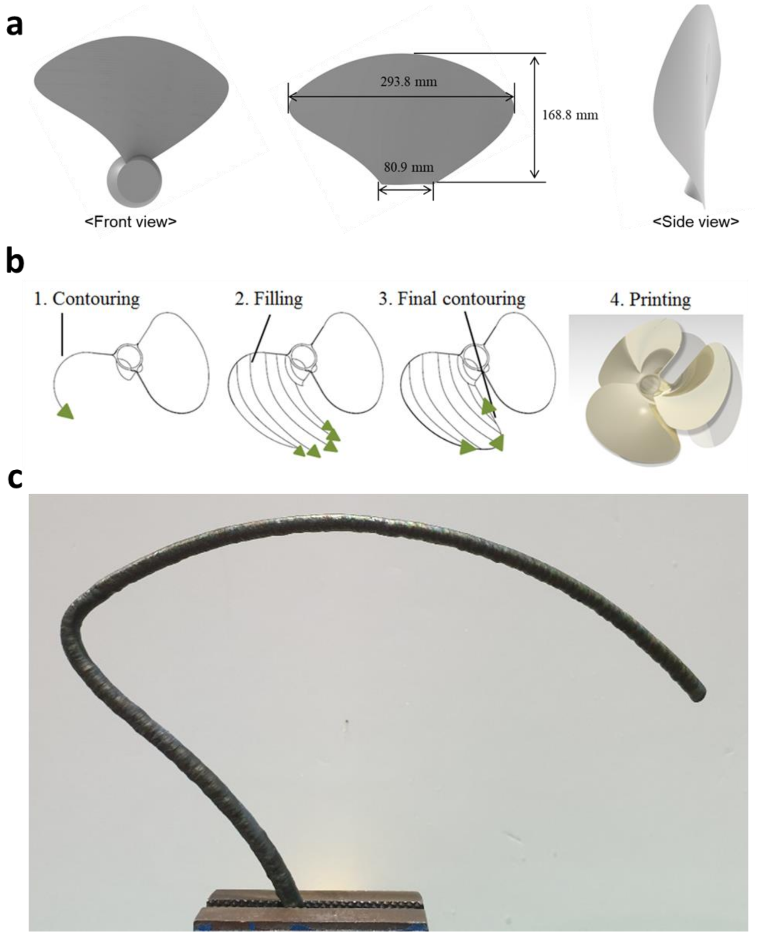


**Figure S12.** Contouring additive manufacturing method of propellers using M3DPen. **a** Design of free-curved propeller. **b** Schematic diagram of propeller using contouring method. **c** Image of propeller using contouring additive manufacturing.

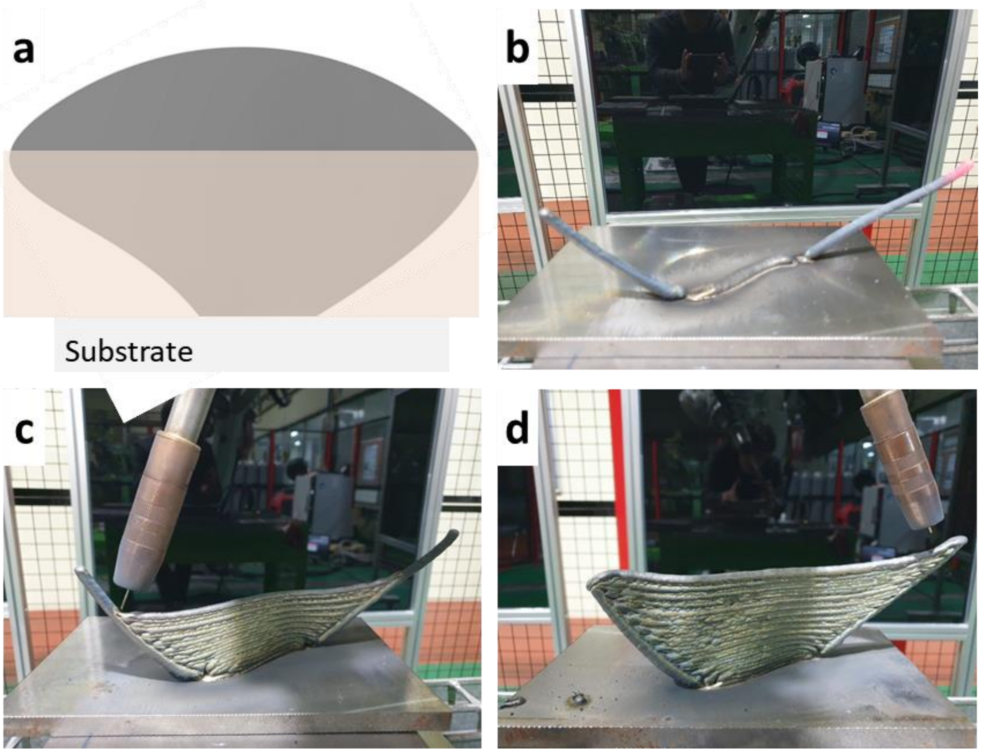


**Figure S13.** Additive manufacturing of propeller using metal supports. **a** design of propeller. **b** Metal support additive manufacturing of propeller. **c-d** Bulk additive manufacturing with zig-zag deposition path.

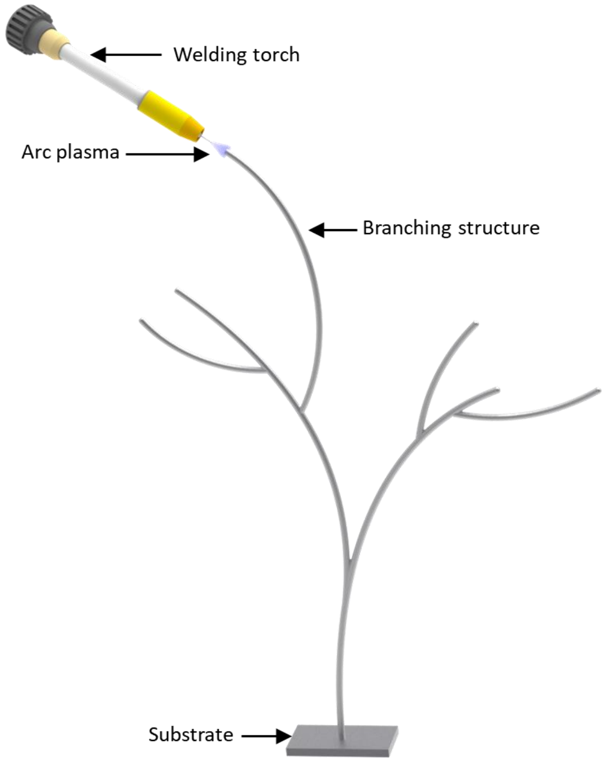


**Figure S14.** Schematic diagram of branching structure using M3DPen.

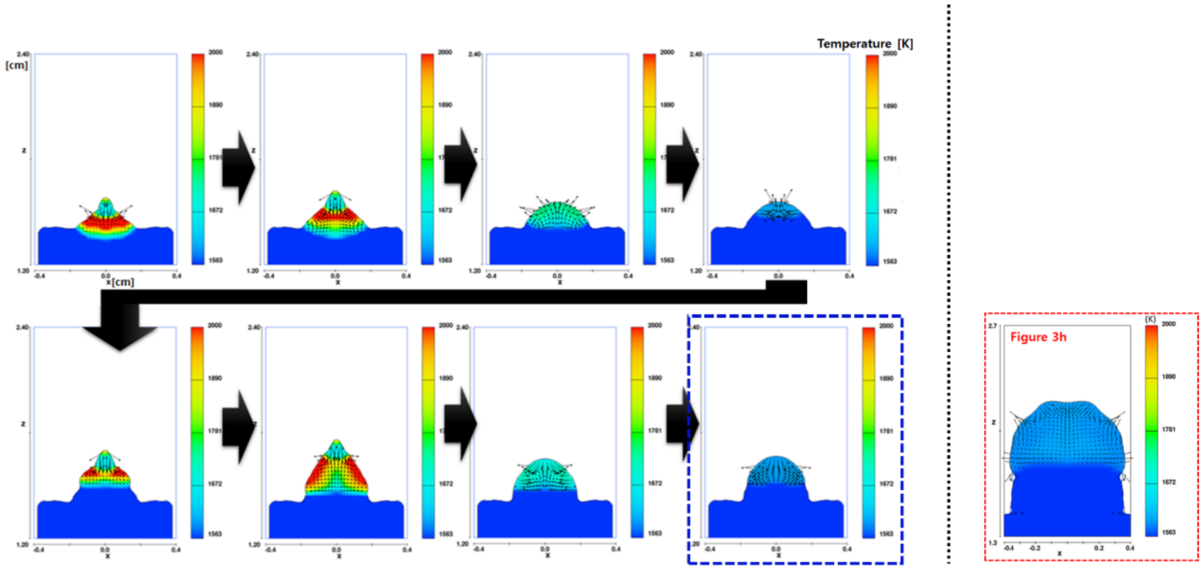


**Figure S15. Smaller diameter formation from metal 3D printing pen process.** This figure describes the M3DPen molten pool behavior using about 30% less wire feed rate and diameter of metal wire than those used in the process of Figure 3. As can be seen from the analysis results, it can be seen that the diameter of the printed structure (dotted-box) can be roughly reduced by half. Therefore, it can be seen that a M3DPen with a smaller diameter can be formed, and a separate optimization process is required to implement the process.

**Table S1** Cross-sectional area data according to the input energy of the metal bead

| Ref | Manuscript | Input Energy (kJ/mm) | Area ($mm^2$) | Speed (mm/s) | Throughput ($mm^3/s$) | Process | Cross section |
|---|---|---|---|---|---|---|---|
| 57 | Experimental optimization of laser additive manufacturing process of single-crystal nickel-base superalloys by a statistical experiment design method | 0.1 | 0.2514 | 15 | 3.771 | | 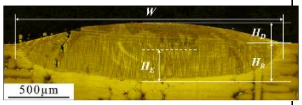 |
| 58 | Causes analysis on cracks in nickel-based single crystal superalloy fabricated by laser powder deposition additive manufacturing | 0.031 | 0.0529 | 8 | 0.4232 | DED | 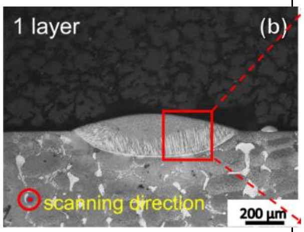 |
| 59 | A new physics-based model for laser directed energy deposition (powder-fed additive manufacturing): From single-track to multi-track and multi-layer | 0.216 | 0.0198 | 3 | 0.0594 | | 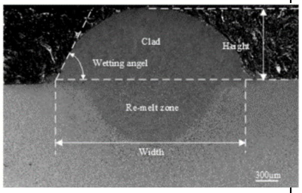 |
| 60 | Multi-beam laser additive manufacturing | 0.0001 | 0.0003 | 600 | 0.18 | | 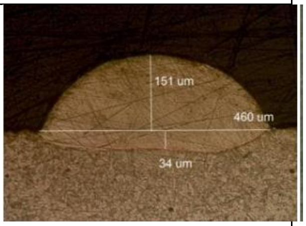 |

| | | | | | | | |
|---|---|---|---|---|---|---|---|
| 61 | Characterization of heat affected zone liquation cracking in laser additive manufacturing of Inconel 718 | 0.5 | 1.245 | 2 | 2.49 | | 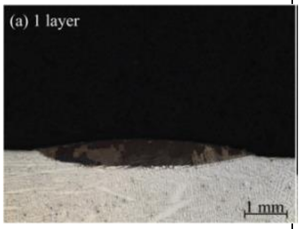 |
| 62 | Modeling of thermal behavior and mass transport in multi-layer laser additive manufacturing of Ni-based alloy on cast iron | 0.15 | 0.240 | 6 | 1.44 | | 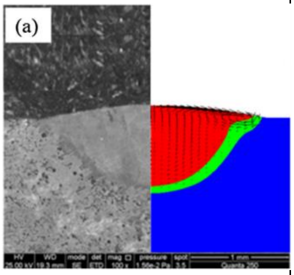 |
| 63 | Melt-pool motion, temperature variation and dendritic morphology of Inconel 718 during pulsed- and continuous-wave laser additive manufacturing: A comparative study | 0.1 | 0.263 | 6 | 1.578 | | 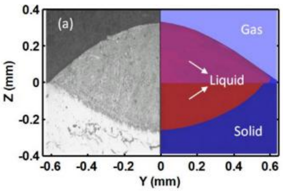 |
| 64 | Direct Energy Deposition - Laser Additive Manufacturing of Titanium-Molybdenum alloy: Parametric studies, microstructure and mechanical properties | 0.269 | 2.813 | 6.67 | 18.76271 | | 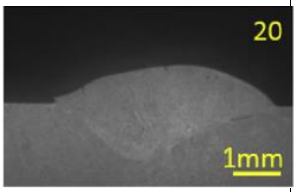 |

| | | | | | | | |
|---|---|---|---|---|---|---|---|
| 65 | On productivity of laser additive manufacturing | 0.034 | 0.0115 | 5 | 0.0575 | | 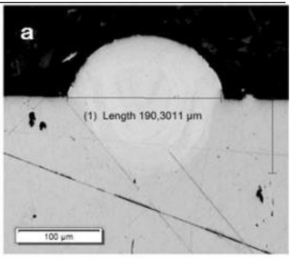 |
| 66 | An analytical model of the melt pool and single track in coaxial laser direct metal deposition (LDMD) additive manufacturing | 0.062 | 0.0029 | 4 | 0.116 | | 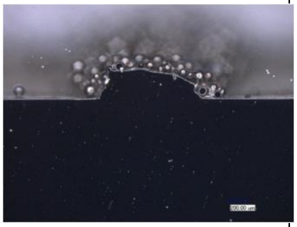 |
| 67 | Laser additive manufacturing of structural-graded bulk metallic glass | 0.015 | 0.743 | 13.33 | 9.90419 | | 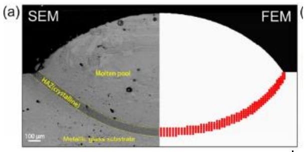 |
| 68 | Working distance passive stability in laser directed energy deposition additive manufacturing | 0.019 | 0.010 | 16.9 | 0.169 | | 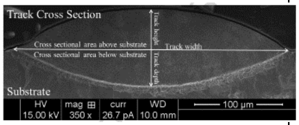 |
| 69 | Observation of keyhole-mode laser melting in laser powder-bedfusion additive manufacturing | 0.00078 | 0.0116 | 188 | 2.1808 | PBF | 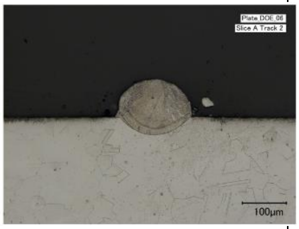 |
| 70 | Development of porous medical implant scaffolds via laser additive manufacturing | 0.00025 | 0.0033 | 600 | 1.98 | | 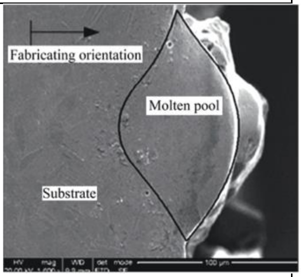 |

| | | | | | | | |
|---|---|---|---|---|---|---|---|
| 71 | Preliminary Investigation of Keyhole Phenomena during Single Layer Fabrication in Laser Additive Manufacturing of Stainless Steel | 0.0005 | 0.0072 | 650 | 4.68 | | 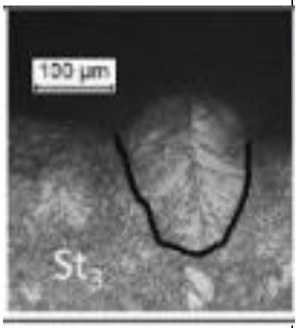 |
| 72 | New Developments of Laser Processing Aluminium Alloys via Additive Manufacturing Technique | 0.02 | 0.0196 | 10 | 0.196 | | 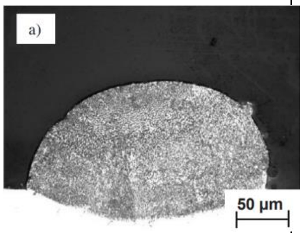 |
| 73 | Single track formation in selective laser melting of metal powders | 0.00025 | 0.0052 | 200 | 1.04 | | 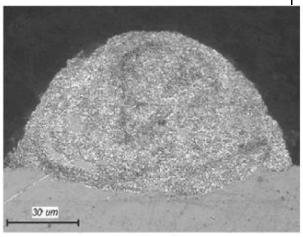 0.20 m/s |
| 74 | Influence of processing parameters on the evolution of melt pool, porosity, and microstructures in Ti-6Al-4V alloy parts fabricated by selective laser melting | 0.00039 | 0.0025 | 500 | 1.25 | | 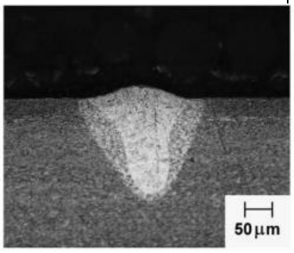 |
| 75 | Laser energy absorption behavior of powder particles using ray tracing method during selective laser | 0.00013 | 0.0073 | 3000 | 21.9 | | 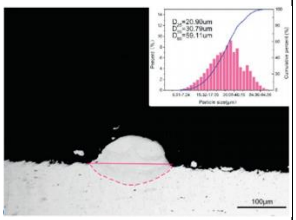 |

| | | | | | | | |
|---|---|---|---|---|---|---|---|
| | melting additive manufacturing of aluminum alloy | | | | | | |
| 76 | Laser Additive Melting and Solidification of Inconel 718: Finite Element Simulation and Experiment | 0.00029 | 0.0272 | 960 | 26.112 | | 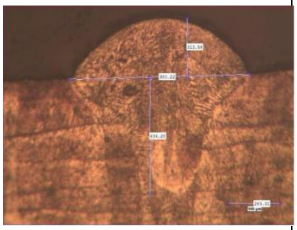 |
| 77 | On the formation of AlSi10Mg single tracks and layers in selective laser melting: Microstructure and nano-mechanical properties | 0.0004 | 0.0034 | 250 | 0.85 | | 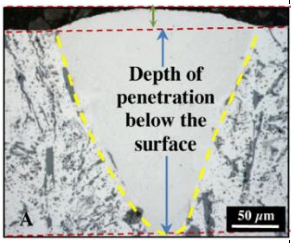 |
| 78 | Melt Pool Characterization for Selective Laser Melting of Ti-6Al-4V Pre-alloyed Powder | 0.000975 | 0.0001 | 200 | 0.02 | | 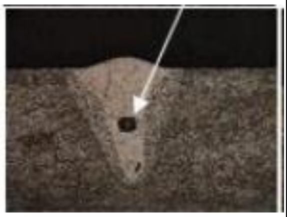 |
| 79 | Selective laser melting of Ti6Al4V alloy for biomedical applications: Temperature monitoring and microstructural evolution | 0.0005 | 0.0002 | 100 | 0.02 | | 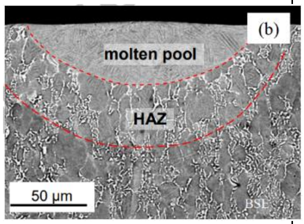 |

| | | | | | | | |
|---|---|---|---|---|---|---|---|
| 80 | Processing AlSi10Mg by selective laser melting: parameter optimisation and material characterisation | 0.00025 | 0.013 | 800 | 10.4 | | 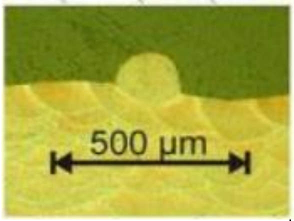 |
| 81 | Gaussian process-based surrogate modeling framework for process planning in laser powder-bed fusion additive manufacturing of 316L stainless steel | 0.0001 | 0.016 | 2500 | 40 | | 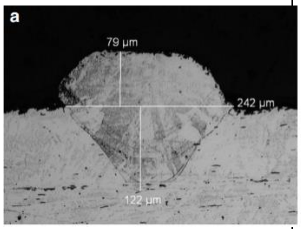 |
| 82 | Selective laser melting of alumina: A single track study | 0.0257 | 0.129 | 11.67 | 1.50543 | | 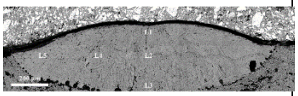 |
| 83 | Analysis of processing parameters and characteristics of selective laser melted high strength Al-Cu-Mg alloys: From single tracks to cubic samples | 0.0008 | 0.002 | 250 | 0.5 | | 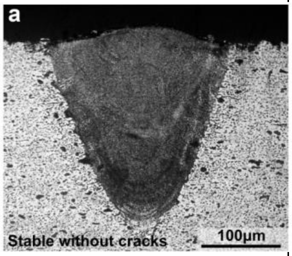 |
| 84 | Single scan track analyses on aluminium based powders | 0.0036 | 0.017 | 50 | 0.85 | | 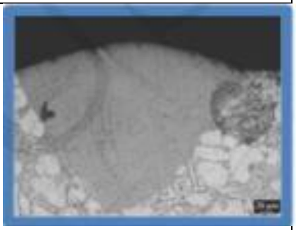 |

| | | | | | | | |
|---|---|---|---|---|---|---|---|
| 85 | Single track and single layer formation in selective laser melting of niobium solid solution alloy | 0.00165 | 0.016 | 200 | 3.2 | | 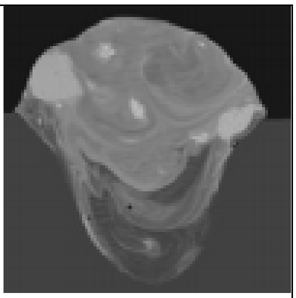 |
| 86 | Determination of the control points for circle and triangle route in wire arc additive manufacturing (WAAM) | 0.88 | 13.599 | 5 | 67.995 | | 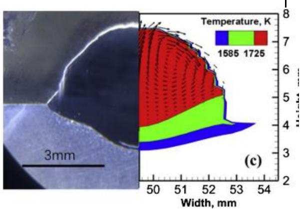 |
| 87 | Investigation of mechanical properties for hybrid deposition and micro-rolling of bainite steel | 0.535 | 11.923 | 10 | 119.237 | | 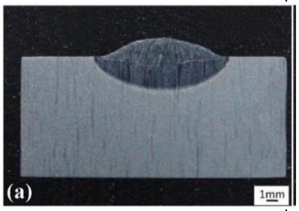 |
| 88 | Numerical analysis of heat transfer and fluid flow in multilayer deposition of PAW-based wire and arc additive manufacturing | 0.828 | 9.920 | 5 | 49.6045 | WAAM | 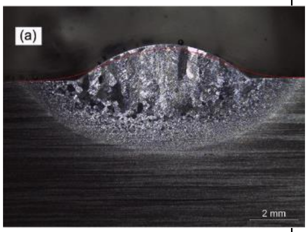 |
| 89 | Three-dimensional numerical simulation of arc and metal transportin arc welding based additive manufactu | 0.484 | 4.873 | 9.5 | 46.29635 | | 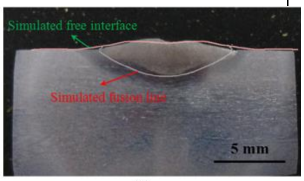<br>(a) |

| | | | | | | | |
|---|---|---|---|---|---|---|---|
| 90 | Forming appearance control of arc striking and extinguishing area in multi-layer single-pass GMAW-based additive manufacturing | 0.716 | 18.827 | 5 | 94.1085 | | 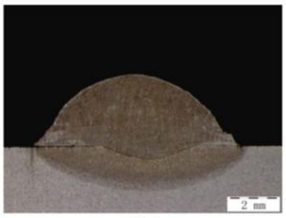 (b) |
| This work | | 0.1308 | 78.5 | 1 | 78.5 | M3DPen | |
